# Emerging Protective Actions of PGC-1*α* in Diabetic Nephropathy

**DOI:** 10.1155/2022/6580195

**Published:** 2022-10-10

**Authors:** Yuqing She, Mei Yu, Liang Wang, Yajing Wang, Penghua Fang, Zhenwen Zhang

**Affiliations:** ^1^Key Laboratory for Metabolic Diseases in Chinese Medicine, Nanjing University of Chinese Medicine, Nanjing 210023, China; ^2^Department of Endocrinology, Pukou Branch of Jiangsu People's Hospital, Nanjing 211899, China; ^3^Department of Endocrinology, Clinical Medical College, Yangzhou University, Yangzhou 225001, China

## Abstract

Renal impairment is affected by various mechanisms of oxidative stress, mitochondrial dysfunction, and basement membrane thickening, which are the major causes of renal dysfunction in diabetes. Of note, hyperglycemia-induced mitochondrial dysfunction has been identified as a common cause of diabetic nephropathy and renal impairment, and the decrease in PGC-1*α* expression brought on by hyperglycemia plays an immensurable role in both the reduction of mitochondrial biogenesis and the rise in oxidative stress. Reduced PGC-1*α* expression levels may occur with rising SGLT2-dependent increase of cytoplasmic sodium and protons in the renal cells of diabetes, even if the precise mechanism of hyperglycemia-induced disruption of PGC-1*α* expression has not been identified. Additionally, it has been observed that SGLT2 inhibitors enhance PGC-1*α* expression and activity and decrease cytoplasmic sodium and protons in many kidney cells, which may be helpful in reducing renal impairment brought on by diabetes. This review summarizes our and other recent studies on the function of PGC-1*α* in diabetic nephropathy, provides another potential mediator of the lower PGC-1*α* expression levels brought on by hyperglycemia in diabetics, and identifies a new pathogenesis of diabetes-related renal impairment. It also explains the mechanism underlying the protective effects of SGLT2 inhibitors on diabetic nephropathy. Therefore, it should be taken into account that SGLT2 inhibitors are an effective therapeutic strategy for reducing renal dysfunction caused by diabetes.

## 1. Introduction

In recent decades, diabetes mellitus (DM) has become a significant healthcare problem of aging, affecting millions of people all over the world [1]. DM affects every cell in the body, which explains the broad range of complications, including accelerated cardiac failure and renal impairment [2]. Diabetic nephropathy is manifested by tubular, interstitial, and arteriolar lesions and abnormalities [3]. Renal impairment is affected by various mechanisms of oxidative stress, mitochondrial dysfunction, and basement membrane thickening, which are the major causes of renal dysfunction in diabetes [4–6]. Notably, diabetic nephropathy and renal impairment have been linked frequently to hyperglycemia-induced mitochondrial dysfunction [6, 7]. There are numerous ways in which modifications might alter the structure and function of mitochondria, including changes to the membrane's integrity, the generation of ROS, the number of mitochondria, and the process of biogenesis [6, 7].

A growing body of research has shown that the reduction in PGC-1*α* expression that leads to abnormalities in mitochondrial biogenesis and ROS generation is directly related to the hyperglycemia-induced mitochondrial dysfunction [8–10]. Numerous studies have shown that diabetes decreases PGC-1*α* expression and activity [10–12]. Additionally, podocytes and renal tubular cells may express less PGC-1*α* when hyperglycemia is present [13–15]. As a result, diabetic patients' renal tubular cells and podocytes have decreased mitochondrial biogenesis and ATP production [13–15]. In return, the elevation of PGC-1*α* expression could promote the mitochondrial biogenesis and ATP production in the renal tubular cells and podocytes of diabetic patients [13–15]. These findings imply that the pathological progression of diabetic nephropathy may be greatly influenced by the reduction of PGC-1*α* expression brought on by hyperglycemia.

This narrative review provides a new mediator of the decreased PGC-1*α* expression levels brought on by hyperglycemia, summarizes our research and that of other recent researchers regarding the function of PGC-1*α* in diabetic nephropathy, and establishes a novel pathogenesis for renal impairment brought on by diabetes.

## 2. The Effect of Oxidative Stress on Renal Impairment and PGC-1*α* Expression

Chronic oxidative stress, which mainly impacts the endocrine and immunological systems, leads to disturbed metabolic homeostasis, which results in the development of diabetes. Subsequent overproduction of reactive oxygen and proinflammatory factors creates a vicious circle in which persistent low-grade systemic inflammation and chronic oxidative stress interact. The development of diabetes-related renal impairment is accompanied by an increase in reactive oxygen, which alters mitochondrial activity and biogenesis in diverse kidney cells [6, 7]. A damaging feedback loop might be created by changes in mitochondrial homeostasis, which would worsen the damage to the kidney's tubular, podocyte, and mesangial cells [6, 7].

Although the exact mechanism of oxidative stress-induced diabetes-associated renal impairment has not been fully analyzed, decreased PGC-1*α* expression may be linked to decreased mitochondrial biogenesis as well as increased oxidative stress, which may exacerbate oxidative stress-induced diabetes-associated renal impairment [13–15]. PGC-1*α* has been connected to thermogenesis and the control of energy metabolism since it was initially discovered as a PPAR-interacting protein in brown adipose tissue [16]. Following that, research has shown that PGC-1*α* enhances both mitochondrial biogenesis and glucose metabolism [17–21]. In fact, PGC-1*α* is a master regulator of mitochondrial biogenesis, coordinating the transcriptional machinery to increase mitochondrial mass, enabling tissue response to rising energetic demands [17–20].

Numerous studies have shown that kidney dysfunction caused by PGC-1*α* deficiency accelerates oxidative stress and causes kidney dysfunction [21, 22]. First, reduced PGC-1*α* expression in the kidneys of diabetic rats has been linked to excessive ROS generation by defective mitochondria [23]. Second, PGC-1*α* has been shown to protect against oxidative stress [21, 22]. According to numerous reports, PGC-1*α* regulates ROS-scavenging enzymes, including the mitochondrial proteins manganese superoxide dismutase 1 and 2 [22–24]. Besides, PGC-1*α*, which can boost mitochondrial electron activity, has been described as a regulator of ROS metabolism by St-Pierre et al. [22]. Notably, PGC-1*α* overexpression lowers ROS production and increases mitochondrial number, biogenesis, and ATP production [21, 23]. In contrast, PGC-1*α* depletion by siRNAs prevents the upregulation of SOD1 and SOD2, as well as glutathione peroxidase 1 (GPX1), another ROS-scavenging enzyme, indicating that PGC-1*α* regulates cytoprotective responses under oxidative stress [22, 25]. Furthermore, PGC-1*α* expression rises in parallel with antioxidant defenses when mouse embryonic fibroblasts are exposed to H_2_O_2_, but PGC-1*α* knockdown suppresses the rise in ROS detoxifying genes in response to H_2_O_2_ [22]. Additionally, a number of studies have suggested that pharmacological PGC-1*α* activation could guard against mitochondrial malfunction and oxidative stress [26–28]. For instance, injection of resveratrol reduced oxidative stress and mitochondrial dysfunction in mouse models of diabetes through the activation of the AMPK/SIRT1/PGC-1*α* axis [26–28].

Taken together, PGC-1*α* can serve as a master regulator of oxidative stress and mitochondrial biogenesis, thus acting as a key regulator of ROS primarily generated by mitochondria.

## 3. The Protective Role of PGC-1*α* in Diabetes-Associated Renal Impairment

### 3.1. Hyperglycemia Inhibits PGC-1*α* Expression in the Kidney

It is well known that PGC-1*α* expression in the kidney is directly influenced by diabetes and experimentally induced hyperglycemia [11–15, 23]. Patients with diabetic nephropathy had lower levels of PGC-1*α* gene expression in their kidney tissue when they were examined in a clinical context [15]. Hyperglycemia-induced PGC-1*α* downregulation has been seen in animal models of diabetic nephropathy. Previous research has demonstrated that diabetic mice and rats have significantly lower levels of PGC-1*α* expression in their kidneys [11–14, 23, 29–32]. Low PGC-1*α* levels were subsequently linked to impaired renal function, glomerulosclerosis, and renal ectopic lipid deposition [23]. Additionally, the podocyte-specific PGC-1*α* knockout animals showed lower mtDNA stability and transcription as well as altered mitochondrial biogenesis machinery and mitochondrial fusion-fission apparatus [33].

In addition to studies conducted in animals, high glucose (HG) significantly decreased PGC-1*α* expression in HK-2 cells and podocytes in vitro [15, 29–31]. Consistently, a high (HG, 25 mM) concentration of glucose medium for 48 hours or 7 days caused a significant drop in PGC-1*α* levels in mesangial cells [23, 32, 33]. Functionally, HK-2 and mesangial cells as well as podocytes showed increased mitochondrial dysfunction and ROS formation in response to high glucose-induced reduced PGC-1*α* [15, 23, 33]. Taken together, these findings suggest that podocytes, tubular cells, and mesangial cells all exhibit hyperglycemia-induced downregulation of PGC-1*α*, which appears to be a common characteristic of diabetic nephropathy and plays a significant pathophysiological role in diabetes-related renal impairment.

On the other hand, PGC-1*α* overexpression or maintenance of PGC-1*α* expression and activity by its activators (resveratrol, PGRN, berberine, Rap1, Tug1, and pyruvate kinase M2) ameliorated renal function deterioration and glomerulosclerosis and decreased mitochondrial ROS generation in tubular and mesangial cells as well as podocytes in vitro and in vivo [23, 29, 34–40]. These findings support the hypothesis that PGC-1*α* may serve as a possible nephroprotective target in renal impairment caused by diabetes.

### 3.2. The Mechanisms of Hyperglycemia-Induced Low PGC-1*α* Expression

#### 3.2.1. The Alteration of AMPK/SIRT1 Pathway under Hyperglycemic Conditions

It has been reported that downregulation of PGC-1*α* protein levels and activity in diabetes has been assumed to be a significant cause or consequence of hyperglycemia [23], with the reason being that hyperglycemia in vivo could produce free radicals and generate oxidative stress [23]. However, the exact mechanisms causing reduced PGC-1*α* expression brought on by hyperglycemia are largely unknown.

**Figure 1:**
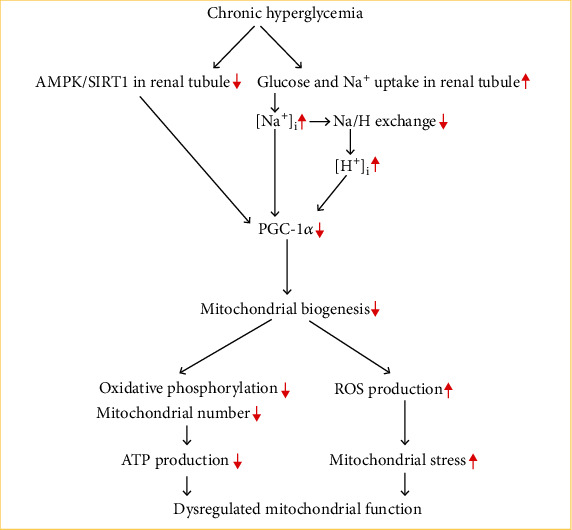
The hyperglycemia-induced mitochondrial dysfunction in diabetes-associated renal impairment. The hyperglycemia-induced elevation of SGLT2 activity results in increased cytosolic sodium and proton and decreased sodium-proton exchanger activity. The SGLT2-dependent elevation of cytoplasmic sodium and protons could cause inhibition of PGC-1*α* expression in renal cells with diabetes. Besides, the hyperglycemia-induced reduction of AMPK/SIRT1 activity could cause inhibition of PGC-1*α* expression in renal cells. Furthermore, low PGC-1*α* expression may be associated with decreased mitochondrial biogenesis as well as increased oxidative stress, potentially exacerbating diabetic nephropathy.

PGC-1*α* activity is influenced by both posttranslational modifications and gene expression levels [10]. It is important to note that the altered AMPK/SIRT1 pathways have been linked to diabetes and have been proposed to explain why PGC-1*α* activity is reduced [41, 42] (see Figure 1). AMPK phosphorylation stimulates the production and activity of PGC-1*α* [41]. SIRT1 deacetylates and activates PGC-1*α*, which prevents mitochondrial biogenesis [42]. An increasing body of research has discovered that hyperglycemia may affect AMPK activity and SIRT1 expression and that the severity of diabetes is negatively connected with AMPK/SIRT1 activity [11, 13, 36, 38]. Furthermore, when subjected to chronic hyperglycemia in diabetes, low AMPK activity and SIRT1 expression levels may decrease PGC-1*α* expression and activity [11, 13, 36, 38, 42]. Additionally, the animals with the podocyte-specific deletion of SIRT1 showed reduced PGC-1*α* expression and activity, as well as elevated albuminuria and mitochondrial damage [43]. Therefore, a decrease in PGC-1*α* activity and mitochondrial dysfunction in the pathophysiology of diabetes-related renal impairment may be attributed to changes in the AMPK/SIRT1 pathways.

#### 3.2.2. Inhibition of Endogenous PGC-1*α* via SGLT2-Dependent Elevation of Cytoplasmic Sodium and Protons under Hyperglycemic Conditions

It has been noted that hyperglycemia lowered glucagon secretion by SGLT2-dependent increase of intracellular sodium and protons, resulting in decreased mitochondrial biogenesis and ATP generation in *α* cells [44]. Other SGLT2-expressing cells from hyperglycemic animals, such as renal tubular cells and cardiomyocytes, have also been found to exhibit intracellular sodium and proton buildup phenomena [44–46]. In contrast, SGLT2 inhibitors decreased cytosolic sodium and protons indirectly by activating the sodium-proton exchanger and sodium-potassium ATPase in diabetes experimental models [44–46]. This suggests that SGLT2-dependent cytosolic sodium and proton reduction in renal tubular cells may be the mechanism by which SGLT2 inhibitors protect renal function in type 2 diabetes [44–46]. Altogether, these findings suggest that hyperglycemia increases cytoplasmic sodium and protons as well as decreases mitochondrial biogenesis and ATP production via SGLT2, which further indicates that hyperglycemia-induced SGLT2-dependent elevation of cytoplasmic sodium and protons could be a new pathogenesis for diabetic nephropathy [44–46].

As mentioned above, the primary aspect of diabetes that causes reduced PGC-1*α* expression is hyperglycemia [10–15, 23]. In addition, type 2 diabetes is characterized by both decreased PGC-1*α* function and increased SGLT2 activity in the diabetic kidney [47]. Furthermore, it has been shown in experimental models of diabetes that elevation of SGLT2 activity brought on by hyperglycemia increases cytosolic proton, sodium, and sodium-proton exchanger activity [44–46]. In contrast, SGLT2 inhibitors raise PGC-1*α* expression and mitochondrial biogenesis in diabetic renal tubular cells while decreasing cytosolic sodium and proton levels [44, 45, 47–49]. We hypothesize that the SGLT2-dependent rise of cytoplasmic sodium and proton could result in a reduction in PGC-1*α* expression in diabetic renal cells since SGLT2 inhibitors have the ability to control cytosolic sodium and proton and PGC-1*α* expression in renal cells of diabetic patients [44, 45, 47–49] (see Figure 1). In short, persistent hyperglycemia causes SGLT2 to be overloaded, which causes an excess of sodium and glucose to be transported and, as a result, an accumulation of cellular Na^+^. The sodium-proton exchanger activity is reduced in response to the rise in intracellular Na^+^, which leads to a buildup of cellular H^+^. As a consequence, acidosis develops as the cytosolic pH values drop. At the same time, insufficient ATP generation prevents cells from maintaining the Na^+^/K^+^-ATPase activity. These pathways contribute to the decrease of PGC-1*α* levels, which ultimately results in excessive ROS generation and mitochondrial oxidative stress. However, the exact mechanisms underlying the association between the low expression of PGC-1*α* in diabetic renal cells and the SGLT2-dependent rise of cytoplasmic sodium and protons remain unknown and require further investigation.

Taken together, these findings suggest that SGLT2-dependent elevation of intracellular sodium and protons might act as a potential mediator of hyperglycemia-inhibited PGC-1*α* expression in diabetic nephropathy.

### 3.3. Endogenous PGC-1*α* Induced by SGLT2 Inhibitors Protects Diabetic Nephropathy

**Figure 2:**
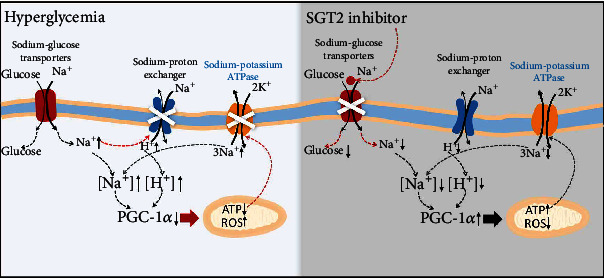
The mechanism underlying the protective effect of SGLT2 inhibitors on diabetes-associated renal impairment. The SGLT2 inhibitors result in decreased cytosolic sodium and protons as well as increased PGC-1*α* expression and mitochondrial biogenesis in the renal tubular cells of diabetes.

The mechanism underlying the positive effect of SGLT2 inhibitors on diabetes-related renal impairment has recently been found to be that they regulate the hyperglycemia-induced PGC-1*α* expression in various cells [47–49] (see Figure 2). According to Guo et al., marein may be able to inhibit the development of diabetic nephropathy and boost PGC-1*α* expression in high glucose-treated HK-2 cells and the renal tissue of db/db mice through inhibiting the expression of renal SGLT2 [48]. In addition, dapagliflozin, an SGLT2 inhibitor, partially reversed the downregulation of PGC-1*α* expression brought on by hyperglycemia in HK-2 cells, in mouse primary renal proximal tubule cells, and in the renal cortex of hyperglycemic mice fed a high-fat diet [49]. Furthermore, SGLT2 gene knockdown with siRNA resulted in an increase in PGC-1*α* expression in HK-2 cells [49]. Moreover, SGLT2 inhibitors reduce the amount of Na^+^ and H^+^ that accumulates within cells [44], which raises intracellular PGC-1*α* levels and promotes mitochondrial biogenesis and ATP production.

The low PGC-1*α* levels were also significantly increased by the SGLT2 inhibitors empagliflozin and canagliflozin in the cardiomyocytes and adipocytes of high-fat diet/streptozotocin-induced diabetic mice and rats [50–53], which was consistent with the effect of SGLT2 inhibitors to reverse hyperglycemia-induced downregulation of PGC-1*α* expression in the kidney. Furthermore, in obese mice, the SGLT2 inhibitor dapagliflozin increased endothelial PGC-1 expression and mitochondrial biosynthesis [54]. In vitro experiments using high glucose- or palmitic acid-induced human umbilical vein endothelial cells revealed that the SGLT2 inhibitor dapagliflozin boosted PGC-1*α* expression and reduced ROS [54, 55]. All of these findings point to the possibility that SGLT2 inhibitors promote PGC-1*α* expression and activity, which in turn might boost mitochondrial biogenesis and ATP production to protect diabetic nephropathy.

## 4. Conclusions

In summary, the review may reveal yet another potential mediator of the lower PGC-1*α* expression levels brought on by hyperglycemia in diabetics. In short, the SGLT2-dependent rise of cytosolic sodium and protons caused by hyperglycemia is the cause of the decreased PGC-1*α* expression in diabetic patients. It will demonstrate a brand-new etiology of mitochondrial malfunction brought on by hyperglycemia in diabetic renal impairment. Additionally, this theory might eventually shed light on a novel etiology of diabetes-related renal impairment as well as the mechanism underlying SGLT2 inhibitors' positive effects on this condition. Therefore, it should be considered that SGLT2 inhibitors are an effective treatment approach for lowering diabetes-related kidney disease.

## Abbreviations

DN: Diabetic nephropathy
FoxO1: Fork head box O1
HOMA-IR: Homeostasis model assessment-insulin resistance
IGT: Impaired glucose tolerance
LDH: Lactate dehydrogenase
IL-6: Interleukin-6
TNF-*α*: Tumor necrosis factor-*α*
PGC-1*α*: Peroxisome proliferator-activated receptor gamma coactivator 1-alpha
HFD: High-fat diet
DM: Diabetes mellitus
ROS: Reactive oxygen species
FFAs: Free fatty acids.
